# Overcoming Barriers in Oncolytic Virotherapy with HDAC Inhibitors and Immune Checkpoint Blockade

**DOI:** 10.3390/v8010009

**Published:** 2016-01-06

**Authors:** Antonio Marchini, Eleanor M. Scott, Jean Rommelaere

**Affiliations:** Infection, Inflammation and Cancer Program, Tumor Virology Division (F010), German Cancer Research Center (DKFZ), Im Neuenheimer Feld 242, 69120 Heidelberg, Germany; eleanor.scott@oncology.ox.ac.uk (E.M.S.); j.rommelaere@dkfz-heidelberg.de (J.R.)

**Keywords:** oncolytic virus, cancer, combination therapy, HDAC inhibitors, immunotherapy, checkpoint immune blockade antibodies

## Abstract

Oncolytic viruses (OVs) target and destroy cancer cells while sparing their normal counterparts. These viruses have been evaluated in numerous studies at both pre-clinical and clinical levels and the recent Food and Drug Administration (FDA) approval of an oncolytic herpesvirus-based treatment raises optimism that OVs will become a therapeutic option for cancer patients. However, to improve clinical outcome, there is a need to increase OV efficacy. In addition to killing cancer cells directly through lysis, OVs can stimulate the induction of anti-tumour immune responses. The host immune system thus represents a “double-edged sword” for oncolytic virotherapy: on the one hand, a robust anti-viral response will limit OV replication and spread; on the other hand, the immune-mediated component of OV therapy may be its most important anti-cancer mechanism. Although the relative contribution of direct viral oncolysis and indirect, immune-mediated oncosuppression to overall OV efficacy is unclear, it is likely that an initial period of vigorous OV multiplication and lytic activity will most optimally set the stage for subsequent adaptive anti-tumour immunity. In this review, we consider the use of histone deacetylase (HDAC) inhibitors as a means of boosting virus replication and lessening the negative impact of innate immunity on the direct oncolytic effect. We also discuss an alternative approach, aimed at potentiating OV-elicited anti-tumour immunity through the blockade of immune checkpoints. We conclude by proposing a two-phase combinatorial strategy in which initial OV replication and spread is maximised through transient HDAC inhibition, with anti-tumour immune responses subsequently enhanced by immune checkpoint blockade.

## 1. Introduction

Oncolytic viruses (OVs) preferentially infect, replicate in, and destroy tumour cells while sparing normal cells. The potential for viruses to be used in the treatment of cancer was first considered in the early 20th century, following anecdotal reports of transient remissions in cancer patients coinciding with their natural contraction of a viral infection. Early pre-clinical and clinical testing began in earnest in the 1950s and 1960s, but limited efficacy together with safety concerns resulted in near-abandonment of the field during the 1970s and 1980s [1]. However, the deeper understanding of both virus and cancer biology that was gained over the years that followed, coupled with relevant breakthroughs in genetic engineering and biotechnology, brought about a resurgence of interest in oncolytic virotherapy. As a result, the past two decades have seen the development of a vast repertoire of OVs with increased potency, specificity and tolerability, leading to the launch of numerous clinical trials evaluating the safety and efficacy of viruses belonging to at least ten different families [2]. The safety profile of OVs is widely regarded as excellent; maximum tolerated doses have rarely been reached and flu- and fever-like symptoms are the main adverse side effects documented in clinical trials thus far [3].

A milestone was achieved in 2005 when H101 (Oncorine; Shanghai Sunway Biotech, Shanghai, China), a recombinant adenovirus (Ad), received regulatory approval in China in combination with chemotherapy for the treatment of head and neck cancer, making it the world’s first OV to be used in the clinic [2]. More recently, talimogene laherparepvec (T-VEC; Amgen, Thousand Oaks, CA, USA), a modified herpes simplex virus (HSV) expressing the immunostimulatory cytokine granulocyte-macrophage colony-stimulating factor (GM-CSF), was evaluated in a randomised phase III trial for the treatment of patients with unresectable metastatic melanoma (ClinicalTrials.gov identifier: NCT00769704) [4]. Clinical benefit was demonstrated: the treatment met its primary endpoint with a 16% durable response rate and increased median overall survival rate by 4.4 months [5]. Based on these results, a Food and Drug Administration (FDA) advisory panel recommended product approval, which was granted in the U.S. on October 2015 under the brand name Imlygic [6]. The European Medicines Agency has also issued a positive opinion on T-VEC [6] and its approval in the European Union is expected in the coming year.

Other clinically advanced OVs include pelareorep (Reolysin; Oncolytics Biotech, Calgary, AB, Canada), a wild-type reovirus [7], which is being tested in combination with chemotherapy in phase III trials in patients with head and neck cancer (NCT01166542). Also at late-stage clinical evaluation is pexastimogene devacirepvec (Pexa-Vec, JX-594; SillaJen Biotherapeutics, San Francisco, CA, USA and Transgene S.A., Strasbourg, France), a thymidine kinase-deletedvaccinia virus (VV) expressing GM-CSF, which has been tested in a randomised phase IIb clinical trial against advanced hepatocellular carcinoma (NCT01387555) [8]. In general, the results of OV trials are promising and warrant further clinical evaluation of this approach. The recent clinical development of OVs is reviewed more completely in refs [3,9,10].

OVs are endowed with a specific oncotropism, which may be natural or engineered. OVs with a natural tropism for cancer cells include rodent protoparvoviruses (PV; family Parvoviridae), myxoma virus (Poxviridae), Newcastle disease virus (NDV; Paramyxoviridae), reovirus (Reoviridae) and Seneca valley virus (Picornaviridae). These viruses are normally non-pathogenic in humans. For efficient completion of their life cycle, they rely on oncogenic signalling pathways and/or the inability of cancer cells to mount effective innate anti-viral immune responses. OVs that have been engineered to replicate preferentially in cancer cells include variants of measles virus (MV; Paramyxoviridae), poliovirus (Picornaviridae) and vaccinia virus (Poxviridae) used in vaccines, and viruses presenting mutations or deletions in key genes required for replication in normal but not in cancer cells, such as Ad (Adenoviridae), HSV (Herpesviridae), VV and vesicular stomatitis virus (VSV; Rhabdoviridae).

Genetic engineering has improved OV safety through attenuation of viral pathogenicity factors, increased virus selectivity for cancer cells at both entry and transductional levels, and enhanced efficacy through the insertion of therapeutic transgenes, such as GM-CSF, into the viral genome [3,11].

## 2. Mechanisms of OV-Mediated Tumour Destruction

**Figure 1:**
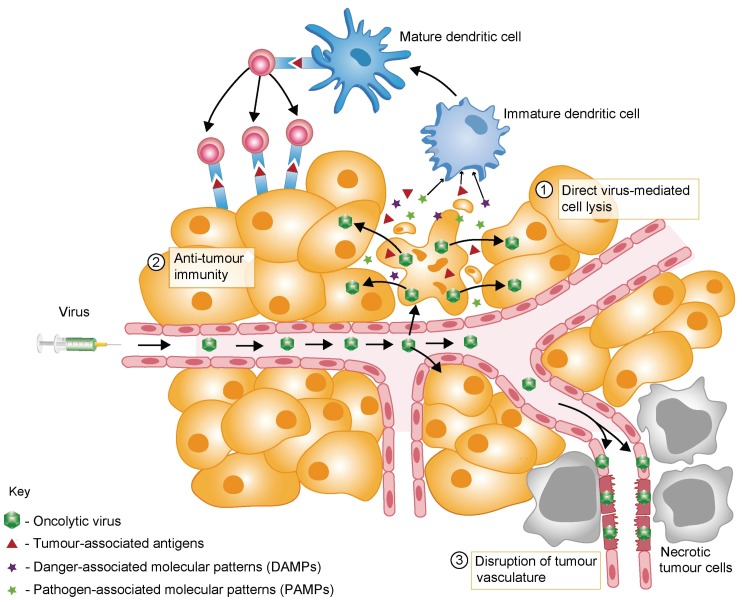
Mechanisms of tumour destruction through oncolytic virotherapy. Oncolytic viruses (OVs) may exert their anti-tumour effects through several mechanisms. OVs induce the death of at least some tumour cells in a direct way by infecting these cells and replicating therein. Progeny virus particles are released and infect neighbouring tumour cells, resulting in amplification of the input OV dose (box 1). OVs often induce an immunogenic cell death (box 2). Tumour-associated antigens, pathogen-associated molecular pattern (PAMPs) and danger-associated molecular patterns (DAMPs) are released from dying tumour cells and come into contact with antigen-presenting cells such as dendritic cells in the tumour microenvironment. Local inflammation, as induced by virus infection, stimulates the maturation of dendritic cells and their migration to draining lymph nodes, where they can present tumour-associated antigens to T cells. Under optimal conditions, this may elicit an anti-cancer CD4^+^ and CD8^+^ effector T cell response that has the potential to kill infected and uninfected tumour cells. In addition, some OVs disrupt the tumour-associated vasculature (via infection of tumour endothelial cells, expression of anti-angiogenic viral proteins and/or OV-induced inflammatory responses), leading to ischemia and necrotic death of uninfected tumour cells (box 3).

In addition to their excellent safety profiles and suitability for genetic modification, OVs are especially promising anticancer agents because they have multiple mechanisms of action (Figure 1).

Virus-induced cell death following infection and in-cell multiplication is a complex, multifaceted process which is triggered mainly by cytotoxic viral proteins, although this has yet to be studied for some oncolytic viruses in use. These proteins have multiple modes of action, which often differ from those activated by conventional cancer therapies. As a result, the likelihood of cancer cells acquiring resistance to OV treatment, as seen with other anti-cancer modalities, has not been documented so far (in contrast to the resistance developed to other anti-cancer modalities). However, it cannot be ruled out that some cancer cells may acquire resistance to OVs through the loss of cellular permissiveness factors, e.g., cell surface receptors that are essential for virus uptake, or the alteration of signalling pathways required for the virus life cycle. Conversely, examples of OVs overcoming cancer cells’ drug resistance have frequently been documented [12]. Following cell lysis, progeny virus particles are released and can infect neighbouring tumour cells. In this manner, repeated cycles of infection, multiplication and lysis may result in viral spread throughout the tumour and self-amplification of the local anti-tumour effect (the direct lytic effect). However, this cycle is likely to be hampered by innate and acquired immune responses of the host as discussed in detail below (see Section 3). Remarkably, tumour-initiating stem-like cells—a subset of cancer cells that are thought to initiate and maintain tumour growth and are generally very resistant to current anti-cancer—have also exhibited sensitivity to oncolytic virotherapies [13].

Although direct viral oncolysis was initially presumed to be the primary mechanism of OV-mediated oncosuppression, there is growing evidence that indirect anti-cancer effects may be critical for OV efficacy. Some OVs, such as VV [14,15] and HSV [16,17], can infect endothelial cells within the tumour bed and thereby induce vascular breakdown, thus indirectly triggering apoptosis or necrosis of uninfected tumour cells.

Of particular relevance for this review, a major component of OV anti-cancer efficacy is now understood to be their induction of anti-tumour immune responses (reviewed in [18,19]). Growing evidence indicates that OV-induced killing of cancer cells often involves immunogenic cell death (ICD), a term used to describe modes of cell death that promote the stimulation of immune responses, generally through the release, secretion and/or surface exposure of danger-associated molecular patterns (DAMPs). Although further studies should be directed towards validating virus-induced ICD in patients, pre-clinical experimentation demonstrates that OVs trigger several forms of ICD, including necrosis, necroptosis, pyroptosis, autophagic cell death and immunogenic apoptosis [20,21]. The predominant mode of death likely depends on the virus, experimental conditions and tumour model studied. Examples of DAMPs mobilization which have been found associated with OV infection of cancer cells include cell-surface exposure of calreticulin (ecto-CRT) (in Ad infections); extracellular release of high mobility group box 1 (HMGB1) (Ad, HSV, MV, VV, and PV); ATP secretion (Ad, VV, coxsackievirus B3, PV); release of heat shock protein (PV); and release of various inflammatory cytokines (MV) (recently reviewed in [21]).

OV-mediated cell killing is also typically associated with the release of a repertoire of pathogen-associated molecular patterns (PAMPs). Characteristic PAMPs include viral components such as nucleic acids (DNA, dsRNA, ssRNA, and 5'-triphosphate RNA), proteins and capsid elements. DAMPs and PAMPs are recognised by pattern recognition receptors on innate immune cells and function as “danger” and “eat me” signals.These promote the maturation of antigen-presenting cells such as dendritic cells, and thereby the activation and expansion of antigen-specific CD4^+^ and CD8^+^T cells in the local tumour-draining lymph nodes (recently reviewed in [21]).

Last but not least, lysis of the tumour cells is accompanied by the release of tumour-associated antigens, which can elicit a potent anti-tumour immune response in the local inflammatory environment established during OV replication. Through this indirect systemic effect, the immune system contributes to the elimination of cancer cells, including those not directly targeted by the OV including those forming small disseminated metastases [9,22] (Figure 1). In particular, various pre-clinical studies have demonstrated the involvement of tumour-specific CD8^+^ T cells and other immune cells in the oncosuppressive activity of OVs [23]. The key role of the immune system in the destruction of cancer cells has also been confirmed at the clinical level for some OVs [24,25,26], sparking a paradigm shift in the field: the immune system, traditionally regarded as a hurdle to effective OV-based therapy because it limits viral replication and spread (see Section 3), may in fact represent its greatest ally.

## 3. Barriers to Successful Oncolytic Virotherapy

**Figure 2:**
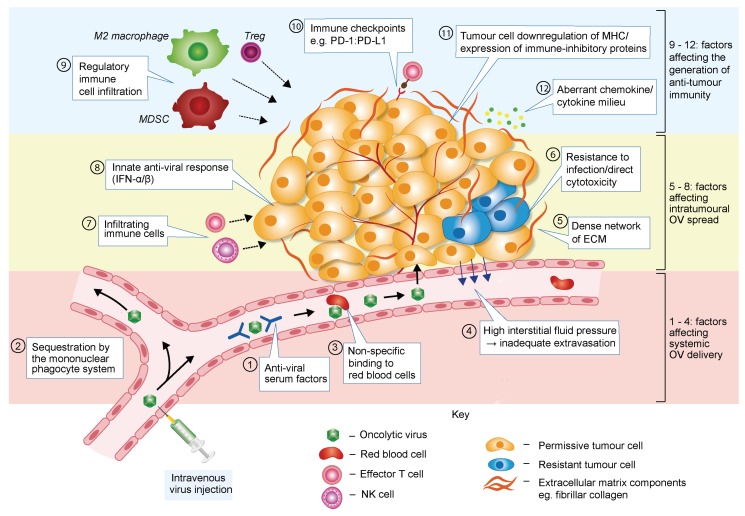
Barriers to effective OV therapy. When delivered to tumours via the bloodstream, oncolytic viruses (OVs) are vulnerable to anti-viral serum factors such as complement proteins and neutralising antibodies (box 1). Binding of these factors accelerates OV clearance from the circulation by macrophages in the liver, lungs and spleen (the mononuclear phagocyte system) (box 2). Systemic delivery of OVs may be further compromised by their non-specific binding to red blood cells (box 3). Once the target site is reached, an additional hurdle is posed by high interstitial fluid pressure within the tumour, which disfavours OV extravasation from the vasculature (box 4). Following extravasation, or after intratumoural OV injection, several factors may limit intratumoural viral spread and therapeutic effectiveness. Abundant extracellular matrix (ECM) and high interstitial fluid pressure represent physical barriers to OV spread by impeding virus diffusion between cells (box 5). Cancer cells within a tumour are likely to be heterogeneous in their susceptibility to virus infection and capacity to support the OV life cycle, with some cells displaying residual intracellular anti-viral activity and/or resistance to OV-mediated cell killing (box 6). Some viruses may also bind normal interstitial cells with a similar affinity as tumour cells, resulting in OV sequestration away from the target cancer cell (not illustrated in the figure). Infiltrating and resident innate immune cells (such as NK cells or macrophages) and anti-viral T cells will also contribute to limit the magnitude of viral production and spread (box 7). In some tumour cells (or normal stromal cells), virus infection may also trigger an anti-viral immune response, leading to release of type I IFN and impediment of virus multiplication (box 8). The immune-tolerant tumour microenvironment may hamper OV-induced anti-cancer immune responses in multiple ways (boxes 9–12), thereby limiting the efficacy of OV-based therapies.

OV-based therapies face various challenges that may weaken their efficacy (Figure 2).

### 3.1. Barriers Limiting Systemic OV Delivery

Despite encouraging results in pre-clinical and clinical studies, further improvement of oncolytic virotherapy is required to maximise fully its therapeutic efficacy. Effective virus delivery is fundamental to successful therapy: sufficient numbers of viral particles must reach tumour sites without first being cleared by the immune system and other physiological filters. In both animal models and clinical trials, OVs are currently administered most commonly by intratumoural injection. However, given the metastatic nature of many cancers and the inaccessibility of some tumour sites, intravenous viral administration arguably represents a more attractive option, with the potential to treat both primary tumours and metastases.

Aspecific binding to serum factors such as pre-immune immunoglobulin M [27], complement [28], anti-viral cytokines [29] and macrophages may result in the rapid neutralisation and clearance of a virus by the reticuloendothelial system [30]. If pre-existing immunity to the virus exists, as is often the case with OVs of human origin, neutralising antibodies may severely hinder systemic delivery [31,32]. Even when there has been no previous exposure to the virus, antibodies can be generated after the first injection, potentially limiting the efficacy of subsequent doses [33]. Elements other than immune factors may also impede intravenous OV delivery. Non-specific binding of OVs to blood cells has been noted in the case of Ad [34], and the characteristically high interstitial fluid pressure within a tumour disfavours extravasation of virions from the tumour vasculature [35].

### 3.2. Barriers Affecting Intratumoural Virus Infection and Spread

The multiplication and intratumoural spread of an OV are critical to the success of OV treatment. Regardless of the route of administration, the virus should ideally achieve a productive infection of cancer cells, resulting in the release of progeny virions which diffuse in the tumour and infect neighbouring cancer cells. This process may be hindered, however, by both the natural resistance of some tumour cells to virus replication or cytotoxicity and the premature destruction of tumour cells that support virus production.

Critical barriers to efficient OV multiplication and spread are raised by the host anti-viral defence and immune responses. When they recognise PAMPs, infected cells activate an innate anti-viral defence process involving the release of type I interferon (IFN) (mainly IFN-α and -β) and other pro-inflammatory cytokines and chemokines. The IFN signalling pathway is often defective in cancer cells [36], preventing them from mounting a robust anti-viral response. However, the residual level of anti-viral activity in some tumour cells may still significantly hinder the efficacy of OV therapy by establishing a microenvironment within and around the infected cell that inhibits virus replication [37,38].

Newly synthesised IFN molecules stimulate the expression of hundreds of genes in an autocrine and paracrine fashion, thereby alerting the immune system to the occurrence of a viral infection. In this way, immune cells (e.g., natural killer (NK) cells, macrophages and virus-specific T cells) are mobilised to eliminate infected cells before virus replication is completed (so-called abortive infection), thus limiting the initial infection and further propagation of the virus.

According to the dynamic equilibrium theory, the kinetics of cancer cell growth and virus spread may have a significant impact on the success of treatment. Tumour cells that have a growth rate faster than the propagation speed of the virus may escape destruction and have a chance to regrow following clearance of the virus by the immune system [39].

Finally, the dense mass of matrix and stromal cells that is characteristic of the tumour microenvironment (TME) can physically impede the movement of virus particles through the tumour [40].

### 3.3. Barriers Hampering OV-Induced Anti-Tumour Immune Response

Tumours can use a plethora of immunosuppressive mechanisms that, like other immunotherapies, may limit the efficacy of OVs.

Tumour immune evasion is mediated to a certain extent by a network of soluble immunomodulatory factors in the TME. Immunomodulatory cytokines such as interleukins [IL]-6, IL-10, and transforming growth factor beta (TGF-β) are expressed by tumour, stromal and/or some infiltrating immune cells, and act most likely in concert to inhibit dendritic cell function, favouring the generation of regulatory over effector T cell responses [41]. Furthermore, tumours may have profound negative effects on the vascular endothelium, hampering T cell adhesion, extravasation and tumour infiltration. For example, tumours can repress the expression of molecules involved in T cell recruitment through the up-regulation of vascular endothelial growth factor [42]. Moreover, a deficiency in appropriate T cell-attracting chemokines, such as CCL2, CCL3, CCL4, CCL5, CXCL9 and CXCL10, may contribute to the inefficient trafficking of effector T cells into the tumour bed [43]. Tumour-derived cytokines can also inhibit effector T cells directly. For instance, TGF-β impairs T cell cytolytic functions [44], whereas IL-10 blocks CD28 co-stimulation of T cells [45]. Other immunosuppressive factors secreted by tumour cells include members of the galectin family—notably galectin-1, which, among other functions, triggers T cell apoptosis [46].

The aberrant chemokine/cytokine make-up of tumours is both cause and consequence of another immunosuppressive feature of the TME: increased infiltration by regulatory cell populations. Regulatory T cells, myeloid-derived suppressor cells and M2-polarised macrophages are actively recruited to the tumour bed and suppress anti-tumour immunity through mechanisms that include the secretion of soluble immunosuppressive factors; direct, contact-dependent inhibition of effector T cell proliferation; and inhibition of antigen-presenting cell function [47,48,49].

Metabolic alterations within the TME, such as hypoxia, nutrient deprivation, abnormal glycolysis and low pH, may have profound negative effects on T cell fitness [42]. For instance, immunosuppressive cell types—as well as tumour cells themselves—may produce amino acid-depleting enzymes such as indoleamine 2,3-dioxygenase and arginase, thereby dampening T cell responses [50,51]. Other tumour cell-intrinsic mechanisms of immune escape comprise the down-regulation of major histocompatibility complex (MHC) molecules and tumour-associated antigens, which precludes recognition by T cells [52]. Moreover, cancer cells often overexpress death receptor ligands (e.g., FasL, TRAIL), triggering the apoptosis of tumour-reactive T cells [52].

Tumours may also avoid immune destruction by usurping immune checkpoints. Critical to the maintenance of self-tolerance, immune checkpoints are a group of inhibitory pathways that dampen the amplitude and duration of immune responses [53]. Two immune checkpoint proteins have garnered particular attention for their roles in cancer: cytotoxic T-lymphocyte-associated protein 4 (CTLA-4) and programmed cell death protein 1 (PD-1). Both are inhibitory receptors expressed by T cells, but the dominant function of each protein differs. CTLA-4 primarily suppresses T cell activation in lymphoid organs by competing with the T cell co-stimulatory receptor CD28 for binding to B7 molecules on antigen-presenting cells, whereas PD-1 dampens T cell effector function in the periphery via its interaction with PD-L1, which is expressed by tumour cells and some immune cells [53]. Both CTLA-4 and PD-1 are up-regulated upon T cell activation and are hence predicted to constitute significant barriers to the success of therapeutics—including OVs—that endeavour to stimulate anti-tumour immunity [54].

To summarise, there are multiple mechanisms of tumour immune suppression which may cooperate or act in parallel to subvert anti-tumour immune responses. As discussed above (Section 2), several OVs have demonstrated a capacity to remodel the immunosuppressive tumour milieu in favour of one that promotes the development of anti-tumour immunity. However, it is increasingly apparent from clinical studies that the anti-tumour immune responses generated by OVs alone are rarely of sufficient potency to induce complete tumour regression. Combining OVs with other agents that combat tumour immune suppression is therefore under investigation as a means to reinforce the immune-mediated component of OV efficacy. One of the most promising strategies is the combination of OVs with immune checkpoints blockade.

## 4. OVs in Combination Therapy

The complexity and heterogeneity of tumours, and their propensity to develop resistance to single-agent treatments, have fuelled research into combination therapy for cancer. The combination of various anti-cancer agents acting through different mechanisms has often improved their efficacy, providing significant clinical benefits [55]. Given the need to improve the efficacy of oncolytic virotherapy in patients, extensive efforts have been directed in recent years to developing strategies that combine OVs with other anti-cancer agents. For the sake of rapid translation from bench to bedside, standard treatment modalities, *i.e.*, chemo- and radiotherapy, have been most widely tested in combination with OVs, resulting in greater therapeutic efficacy in several cases [56,57]. Some of these combinations are currently under assessment in clinical trials [56,57,58], with significant clinical responses already reported [59].

Our increased knowledge of the factors limiting oncolytic virotherapy now permits a more rational selection of drugs which may enhance OV efficacy. This approach is supported by recent studies showing that synergistic anti-cancer effects can be achieved through rationally designed therapies combining OVs with other anti-cancer agents. The wide repertoire of novel anti-cancer therapeutics developed thus far ranges from signalling pathway inhibitors [56], to epigenetic modulators [60], TME modifiers and various forms of immunotherapy [20] thereby providing a number of different strategies with which to manipulate, and hopefully increase the success of oncolytic virotherapy. Combination regimens under investigation aim to: (i) improve systemic OV delivery [9]; (ii) enhance intratumoural OV spread by increasing viral entry, replication or diffusion between neighbouring cells [9]; (iii) augment direct OV-mediated cytotoxicity [9]; and (iv) enhance the anti-tumour immune response elicited during OV therapy [61].

In the following sections, we start by discussing the use of histone deacetylase inhibitors (HDACIs) to overcome some of the barriers limiting virus infection, replication and propagation within the tumour. We then turn to attempts to combine OVs with immune checkpoint blockade antibodies in order to maximise the virus-induced anti-tumour immune response. We conclude by presenting a rationale for a two-phase combinatorial treatment in which OVs are first combined with HDACIs and then with inhibitors of immune checkpoints.

## 5. OVs in Combination with Histone Deacetylase Inhibitors

By affecting the activities of histones and numerous other proteins—including transcription factors, chaperones and regulators of DNA repair, replication and transcription—histone deacetylases (HDACs) have a powerful influence over virtually all cellular processes. HDAC deregulation has been implicated in the promotion of both carcinogenesis and tumour progression (reviewed in [62]), thereby prompting the development of a number of HDACIs with wide-ranging anti-cancer properties [63]. Indeed, HDACIs induce growth arrest, differentiation, senescence and death of cancer cells but not of normal cells.

The mechanisms underlying this onco-selectively are not fully understood [62]. HDACI-induced cell death is often immunogenic, leading to enhanced anti-cancer immune responses [64,65]. Moreover, HDACIs have been found to inhibit angiogenesis [66]. To date, three HDACIs have received FDA approval: vorinostat (Zolinza; Merck, Kenilworth, NJ, USA) for the treatment of cutaneous T cell lymphomas; romidpsin (Istodax; Celgene, Summit, NJ, USA) for the treatment of cutaneous T cell lymphomas and peripheral T cell lymphoma; and belinostat (Beleodaq; Spectrum Pharmaceuticals, Henderson, NV, USA), for the treatment of recurrent or refractory peripheral T cell lymphoma. At least 12 other HDACIs are under clinical investigation as anti-cancer agents, either as monotherapies or in combination with other anti-cancer agents, against a broad spectrum of haematological malignancies and solid tumours [67].

**Figure 3:**
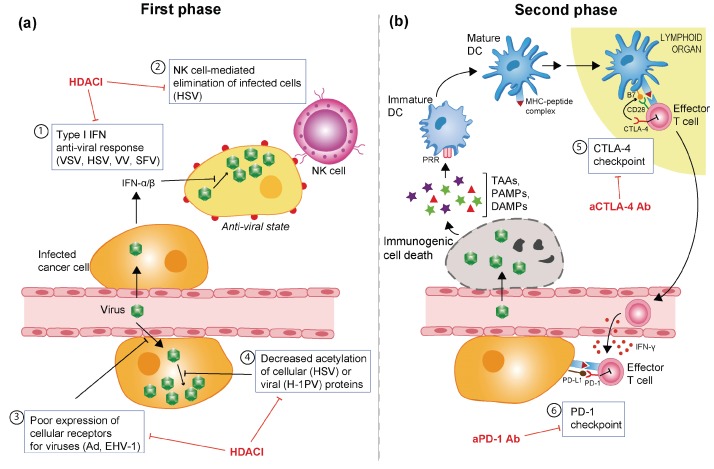
Combinatorial approaches to overcome barriers to OV-induced oncolysis and immune stimulation. (**a**) Multilevel stimulation of OV infection and multiplication by histone deacetylase inhibitors. The combinatorial use of HDACIs clears some of the hurdles (boxes 1–4) that limit the initial phase of the oncolytic virus (OV) oncosuppression process, *i.e.*, virus infection and replication culminating in oncolysis and spread. OVs for which such stimulations have been reported are indicated in brackets (see main text) (**b**) Unmasking induced anti-tumour immunity by means of immune checkpoint inhibitors. As a result of a successful first phase of virus multiplication and tumour cell lysis (panel A), immune cells are activated and can target the tumour in a subsequent step of OV oncosuppression. This bystander effect is, however, counteracted by immune checkpoints acting in lymphoid tissues (box 5) or at the tumour site (box 6). In addition to its IFN-γ-mediated activation by effector T cells, the PD-L1/PD-1 checkpoint can be engaged as a result of OV infection of target tumour cells. The combined administration of immune checkpoint inhibitors, in particular specific antibodies (Ab), is intended to potentiate the immune stimulation achieved by OVs. Ad: Adenovirus; HSV: herpes simplex virus; EHV-1: equine herpesvirus type 1; H-1PV: H-1 parvovirus; SFV: Semliki Forest virus; VSV: vesicular stomatitis virus; VV: vaccinia virus; Ab: antibody; CD28: cluster of differentiation 28; CTLA-4: cytotoxic T-lymphocyte-associated protein 4; DAMPs: danger-associated molecular patterns; DC: dendritic cells; IFN: interferon; MHC: major histocompatibility complex; NK: natural killer cells; PAMPs: pathogen-associated molecular patterns; PD-1: programmed cell death protein 1; PD-L1: programmed death-ligand 1; PRR: pattern recognition receptor; TAAs: tumour-associated antigens.

In addition to their anti-neoplastic effects, HDACIs are known to weaken the cellular anti-viral immune response by impairing the expression of IFN and IFN-inducible genes [68,69,70]. Several groups have therefore explored the possibility of combining OV-based therapy with HDACIs in an effort to suppress the residual anti-viral activity of tumour cells and, as a result, improve OV replication and spread. However, as discussed below, the pleiotropic effects of HDACIs may benefit OV therapy through multiple, and sometimes unexpected, mechanisms (Table 1 and Figure 3).

**Table 1:** OVs in combination with histone deacetylase (HDAC) inhibitors.

Virus	Viral Variant	HDACI(s)	HDAC Selectivity	Cancer Type(s)	*In vivo* Model (Route of OV Delivery)	Mode of Action	Ref.
VSV	VSVΔ51	Vorinostat, MS-275	Classes I and II (Vorinostat)	Various solid tumours	Athymic nude mice (IT or IP)	↓ IFN and IFN-responsive gene expression; ↑ virus multiplication; ↑ intrinsic apoptosis	[71]
			Class I (MS-275)				
	VSVΔ51	Vorinostat	Classes I and II	Prostate cancer	-	↑ NF-κB activity; ↑ autophagy; ↓ IFN-mediated response; ↑ viral replication and apoptosis	[72]
HSV-1	G47Δ	TSA	Classes I and II	Glioma and colorectal cancer	Athymic nude mice (IT)	↓ VEGF secretion; ↓ angiogenesis; ↓ cyclin D1	[73]
	rQNestin34.5	VPA (pre-treatment)	Classes I and IIa	Glioma	Athymic nude mice (IT)	↓ IFN-inducible gene expression; ↑ viral replication	[74]
	R849	TSA	Classes I and II	Oral squamous cell carcinoma	-	↑ NF-κB activity; ↑ viral replication; ↑p21→G1 cell cycle arrest	[75]
	rQNestin34.5	VPA	Classes I and IIa	Glioma	Athymic nude mice (IT)	↓ Innate immune responses; ↓ NK cell activity, through inhibition of STAT5/T-BET signalling	[76]
	ΔICP34.5	Various	-	Breast cancer	-	↑ Viral replication	[77]
EHV-1	Wild type (WT)	VPA (pre-treatment)	Classes I and IIa	Glioma	-	↑ Viral entry	[78]
Ad	Ad5.CMV-LacZ	Romidepsin	Class I	Various solid tumours	-	↑ Viral entry receptors	[79]
	OBP-301	Romidepsin	Class I	Non-small cell lung cancer	-	↑ Viral entry receptors	[80]
	Ad5.CMV-GFP	Romidepsin	Class I	Melanoma	Athymic nude mice (IT)	↑ Viral entry receptors	[81]
	Delta24-RGD	Scriptaid, LBH589	Class I (Scriptaid)	Glioma-initiating stem-like cells	-	↑ Cell death pathways	[82]
			Classes I and II (LBH589)				
VV	VVdd	TSA	Classes I and II	Various solid tumours	Immunocompetent C57BL/6 mice (IV)	↓ IFN-response; ↑ viral replication and spread	[83]
	Western Reserve	TSA	Classes I and II	Various solid tumours	-	↑ Viral replication	[83]
	Western Reserve B18R-TK-Luc+	TSA	Classes I and II	Various solid tumours	Athymic nude mice (IV)	↑ Viral replication	[83]
H-1PV	WT	VPA, sodium butyrate	Classes I and IIa (VPA)	Cervical and pancreatic carcinomas	Athymic nude rats and NOD/SCID mice (IT)	↑ Acetylation and activity of viral effector protein; ↑ virus multiplication; ↑ oxidative stress	[84]
			Classes I and IIa (sodium butyrate)				
SFV	WT	Vorinostat, MS-275	Classes I and II (Vorinostat)	Breast cancer	-	↑ Viral replication and spread	[71]

### 5.1. Vesicular Stomatitis Virus

VSV∆51 is a naturally occurring VSV variant containing a deletion within the *M* gene [85]. This deletion renders the virus incapable of counteracting anti-viral IFN responses in normal cells. In contrast, VSV∆51 replication and lytic activity should occur in cancer cells with defective IFN signalling. However, some cancer cells possess residual anti-viral IFN activity which may impair VSV∆51 infection, replication and spreading. With the aim of overcoming this constraint, VSV∆51 was tested in combination with the HDACIs vorinostat and MS-275 in prostate cancer-derived cell lines and primary human tumour tissue specimens [71]. By inhibiting the expression of IFN and IFN-inducible genes, such as *IRF3*, *IRF7* and *MXI*, both HDACIs enhanced VSV∆51 multiplication and activation of the intrinsic apoptotic pathway, leading to a synergistic induction of cancer cell death. The VSV∆51/MS-275 co-treatment was validated *in vivo* by using several xenograft models of human prostate, colon, ovarian and breast cancer: enhanced virus replication and oncolytic activity within the tumour were confirmed, especially in models originally refractory to VSV treatment [71]. These events were also accompanied by vascular shutdown, leading to a significant reduction of the blood flow through the tumour mass. Remarkably, the boosting effect of MS-275 on virus replication was dependent on the continuous administration of this compound and vanished as the drug was withdrawn. These results provided first evidence that by transiently blocking the IFN response, HDACIs may function as reversible switches to control the extent of virus replication within the tumour. The enhancement of VSV oncolysis by vorinostat in prostate cancer cells was traced back to the reversible induction of nuclear factor kappa B (NF-κB) signalling through increased acetylation, nuclear translocation and DNA binding activity of the NF-κB subunit RELA/p65. The resulting induction of NF-κB-dependent autophagy led to suppression of the IFN response and subsequent enhancement of VSV replication and apoptosis [72]. Furthermore, Bridle and colleagues demonstrated that in the context of a prime-boost vaccination regimen, HDACIs may have advantageous immunomodulatory effects besides the mere inhibition of the innate defence response [86]. In a syngeneic mouse model of intracranially implanted B16-F10 melanoma cells, tumour-bearing animals were treated first with a recombinant Ad expressing the melanoma-associated antigen dopachrome tautomerase and subsequently with an oncolytic VSV expressing the same antigen in the presence or absence of MS-275. MS-275 co-treatment led to a differential immunosuppression in which regulatory and naïve T cells were reduced without compromising the secondary response towards the TAA. This environment improved the functionality of anti-tumour CTLs and resulted in significantly prolonged survival of HDACI-treated animals, relative to those receiving virus alone [86].

### 5.2. Herpesvirus

HSV anti-cancer activity is also potentiated by HDACIs through multiple mechanisms, depending on the HDACI used. Otsuki *et al.* studied the interaction between rQNestin34.5 and valproic acid (VPA) in glioma-derived cell lines [74]. rQNestin34.5 is an oncolytic HSV-1 variant in which the *RL1* gene, encoding the viral virulence factor ICP34.5, is under the control of the glioma-specific nestin promoter [87]. VPA is an HDACI used already in the clinic as an anti-epileptic agent. VPA pre-treatment suppressed the transcription of IFN-stimulated anti-viral genes such as signal transducers and activators of transcription 1 (*STAT1*), protein kinase R (*PKR*) and promyelocytic leukemia (*PML*), thereby enhancing HSV gene expression, propagation and cytotoxicity. Interestingly, these beneficial effects were observed only when VPA was applied prior to, but not concurrently with, administration of the virus, indicating that the timing of VPA treatment is critical for effective combination therapy. *In vivo*, a 10-fold increase in viral titre in the brains of glioblastoma multiforme (GBM)-bearing mice was observed in animals pre-treated with VPA and treated with HSV, resulting in a significant extension of their survival time [74]. It was later discovered that, in addition to impairing the cellular anti-viral response, VPA augments oncolytic HSV therapy by causing a transient decline in immune cell recruitment and activation, with a particularly profound suppressive effect on virus-directed NK cell activity. In particular, VPA inhibited NK-cell mediated cytotoxicity both by down-regulating granzyme B and perforin and by abrogating NK cell-dependent production of IFN-γ through the inhibition of the STAT5/T-BET pathway [76].

The HDACI trichostatin A (TSA) enhanced the replication and oncolytic activity of the ICP34.5-deficient HSV-1 variant R849 in oral squamous carcinoma cells [75]. By increasing the acetylation level of the p65 subunit of NF-κB, TSA promoted the nuclear translocation and activity of NF-κB. This activation led not only to increased viral gene expression but also to an up-regulation of the cyclin-dependent kinase inhibitor p21, resulting in G1 cell cycle arrest and an enhanced anti-tumour effect [75]. Similarly, synergism between TSA and another oncolytic HSV, the multi-attenuated HSV-1 mutant G47∆, was observed in animal models of GBM and colorectal cancer, with the enhanced anti-tumour activity being ascribed, at least in these cases, to cyclin D1 blockade and VEGF inhibition [73]. Recently, a panel of HDACIs have been tested *in vitro* for their ability to increase the replication of ICP34.5-deleted oncolytic HSV-1 in breast cancer-derived cell lines. Pan-HDAC inhibitors or HDACIs targeting class I HDACs were found to be more effective than those inhibiting class II HDACs or those that are selective for a particular HDAC [77].

Equine herpesvirus type 1 (EHV-1) has also demonstrated oncolytic activity against GBM cells, with MHC-1 being one of the receptors used by the virus to enter these cells [78]. Pre-treatment (but not co-treatment) of moderately susceptible GBM cell lines with VPA improved EHV-1 infection and cell-to-cell spread, leading to a synergistic enhancement of oncolytic activity [88]. The sensitising effect of VPA was traced back to the stimulation of EHV-1 entry. As VPA is known to enhance the expression of MHC-1 [89], it has been hypothesised that VPA-mediated up-regulation of MHC-1 may be responsible for the observed increase in viral infection and cytotoxicity [88].

### 5.3. Adenovirus

Combination of Ad with HDACIs also represents a very attractive strategy for improving both Ad-based cancer gene therapy and oncolytic adeno-virotherapy. The most widely used adenoviruses are those derived from serotype 5 (Ad5). Ad5s infect host cells through the cellular coxsackievirus and adenovirus receptor (CAR) and α_v_integrin, which mediate viral surface attachment and internalisation, respectively. Although widely expressed on epithelial cells, CAR is often down-regulated in cancer cells, which hampers the infectivity and consequently the anti-tumour efficacy of Ad. A number of HDACIs, including romidepsin (also named FR901228 or depsipeptide), TSA, sodium butyrate and VPA, have been reported to enhance infectivity and transduction capacity of Ad-based gene transfer vectors by increasing the expression of CAR and α_v_integrin in various solid or haematological cancer-derived cell lines *in vitro* [79,81,90,91,92,93,94] and *in vivo* [81,92]. HDACI-induced overexpression of CAR and α_v_integrin appears to occur preferentially in cancer cells and therefore should not increase the off-target effects of Ad-based treatments in normal cells [90,92,93]. Furthermore, HDACIs can enhance Ad5-TRAIL anti-cancer efficacy by increasing both the transfer and the transcription of the *TRAIL* gene [95], activating various stages of the TRAIL-mediated apoptotic pathway [96,97,98] and reducing expression of the anti-apoptotic proteins Bcl-XL and c-FLIP [99].

In agreement with these results, the anti-cancer activity of oncolytic adenoviruses also proved to benefit from combination with HDACIs. Ad OBP-301 (telomelysin) is a conditionally replicating adenovirus in which the expression of early genes *E1A* and *E1B* is under the control of the telomerase reverse transcriptase promoter. Combination with romidepsin increased Ad OBP-301 infectivity in human non-small cell lung cancer cell lines through the up-regulation of CAR expression, resulting in a synergistic oncolytic activity [80].

Delta24-RGD is an engineered Ad5 variant which replicates preferentially in cancer cells due to both a 24-base pair deletion in the *E1A* gene (pRB binding domain) and the insertion of an arginine-glycine-aspartic acid retargeting peptide into the viral fibre knob domain. This peptide recognises the αvβ3 and αvβ5 integrins, which are often overexpressed in cancer cells. It has recently been reported that the HDACIs scriptaid and LBH589 increased the infectivity of Delta24-RGD in a subset of patient-derived, glioma-initiating stem-like cell cultures that are normally refractory to Delta24-RGD infection, leading to enhanced oncolytic effect [82].

### 5.4. Vaccinia Virus

*In vitro* experiments using various cancer cell lines showed that the oncolytic activity of VV can be potentiated by HDACIs [71]. This was further investigated by MacTavish and colleagues, who tested a subset of HDACIs in combination with attenuated, tumour-selective VV variants harbouring deletions of both the thymidine kinase and vaccinia growth factor genes [100] or the B18R-gene [101] in cancer cell lines resistant to VV infection [83]. Among the HDACIs, TSA was the most effective at increasing VV replication, spread and killing activity. Moreover, while pre-treatment with IFN protected cancer cells from VV infection, TSA was able to rescue virus infectivity, suggesting that the HDACI enhances VV replication in tumour cells mainly through inhibition of the IFN anti-viral response. Notably, TSA was unable to counteract the IFN-mediated anti-viral response in normal cells, in keeping with the specificity of the co-treatment for cancer cells. The co-treatment was validated in a B16F10LacZ metastatic model of lung cancer in which combining TSA with VV decreased the number of lung metastases compared with either agent alone. The enhanced anti-cancer activity of the combination therapy was also confirmed using an HCT116 colon tumour xenograft model. The stimulation of oncosuppression was accompanied by an increase in virus replication in tumours but not in normal tissues. When combined with a VV variant harbouring deletions in the *B18R* gene encoding an IFN-scavenging protein, TSA co-treatment achieved even greater induction of cytotoxicity, presumably owing to the higher sensitivity of this virus to the cellular IFN response. However, the fact that treatment with VV with intact *B18R* still received significant benefit from HDAC inhibition suggests that other uncharacterised mechanisms may underlie the improved cytotoxicity observed [83].

### 5.5. H-1 Parvovirus

The oncolytic activity of H-1PV [102] against cervical and pancreatic carcinoma cell lines can be enhanced by sub-lethal doses of VPA [84]. Strikingly, in rodent xenograft models of cervical and pancreatic carcinoma, H-1PV/VPA combination therapy induced complete and long-lasting tumour remission in all co-treated animals with no evident adverse side effects [84]. Combination therapy was associated with enhanced virus replication and cytotoxicity, relative to H-1PV monotherapy. For the first time, the synergistic effect was attributed to increased acetylation and functional activation of a viral product, namely the replication and cytotoxic NS1 protein. Although the molecular mechanisms underlying the synergistic killing activity were not fully elucidated, evidence was provided that oxidative stress may play an important role. Indeed, both H-1PV and VPA induced the accumulation of reactive oxygen species, leading to increased DNA damage and apoptosis. Therefore, the co-treatment may have caused reactive oxygen species levels to reach a point where the antioxidant capacity of the cell was overwhelmed, thus potentiating apoptosis [84]. The fact that a sub-lethal dosage of VPA—compatible with its current clinical use—lowered the effective therapeutic dose of H-1PV warrants future clinical evaluation of the protocol.

### 5.6. Semliki Forest Virus

It has been reported that both MS-275 and vorinostat enhanced the oncolytic activity of Semliki Forest virus in 4T1 breast carcinoma cells by dampening the anti-viral innate immune response [103].

## 6. Potentiating OV-Elicited Anti-Tumour Immune Responses with Immune Checkpoint Inhibitors

Exciting data show that the tumour-mediated repression of T cell responses can be mitigated through blockade of immune checkpoint proteins, e.g., using antibodies against CTLA-4 or PD-1/PD-L1. The use of these inhibitors as standalone therapies led to durable clinical responses and, in a fraction of patients, long-term remission. On the basis of this important survival benefit, three antibodies—targeting CTLA-4 (ipilimumab) and PD-1 (nivolumab and penbrolizumab)—have been approved by the FDA and are currently the standard therapeutics against advanced metastatic melanoma [42]. A series of other immune checkpoint inhibitors are progressing through early- and late-phase clinical assessment with promising results [104]. Despite the fact that checkpoint blockade therapy is often associated with a number of immune-related adverse side effects due to non-specific immunological activation, the unprecedented durability of the response in some melanoma patients (up to 10 years in the case of ipilimumab [105]) justifies unequivocally the addition of immune checkpoint inhibitors to the current anti-cancer arsenal.

Nonetheless, clinical efficacy is not always achieved. While the 3-year overall survival rate in melanoma patients receiving ipilimumab is less than 20% [105], the anti-tumour effects of checkpoint inhibitors have been even more limited for non-melanoma cancers [106]. These data indicate that a number of tumours have an intrinsically non-immunogenic microenvironment. Although predictive biomarkers of responsiveness are still lacking [54], there is clinical evidence that patients harbouring immunogenic tumours, characterised by pre-existing lymphocyte infiltration among other features, will benefit most from immune checkpoint blockade [107]. This situation calls for the development of appropriate strategies which combine immune therapeutics with agents able to convert a non-immunogenic TME into an immunogenic one [54]. Owing to their ability to induce ICD [21], OVs appear to be able to induce this conversion and create the conditions necessary for effective T cell priming and activation [19]. Therefore, OVs may be ideal candidates to complement immune checkpoint inhibitors such as anti-PD-L1 and anti-CTLA-4 antibodies and thus improve the clinical outcome of these agents. As reported below, recent studies, confirmed that the immunostimulatory properties of OVs can be steered to improve the efficacy of immune checkpoint blockade.

### 6.1. Pre-Clinical Studies

Intraperitoneal administration of a recombinant VSV variant targeted to Her2/neu-expressing tumours, followed by systemic CTLA-4 blockade one day later, elicited a potent anti-tumour CD4^+^ and CD8^+^ T cell response in immunocompetent mice bearing Her2/neu-positive D2F2/E2 murine mammary tumours. A complete and long-lasting remission was achieved in the majority of co-treated animals [108]. Importantly, most surviving animals were resistant to re-challenge with syngeneic parental D2F2 cells not expressing Her2/neu, indicating the development of long-term immunity to tumour antigens [108].

Other compelling evidence has been provided in a recent study by Zamarin and colleagues who combined NDV with systemic CTLA-4 blockade in a bilateral B16-F10 murine melanoma model [23]. When administered singly, NDV replicated in only the injected tumour site, but triggered the infiltration of tumour-specific lymphocytes in both injected and non-injected (distant contralateral) tumours. However, T cells were found to overexpress CTLA-4. This immunosuppressive TME reduced NDV treatment efficacy: despite delayed tumour growth, complete tumour eradication and long-term survival were observed in only ~10% of animals treated with NDV alone [23]. The combination of NDV with CTLA-4 blockade potentiated the systemic anti-tumour immune response, resulting in improved long-term survival of most co-treated animals. This effect was strongly dependent on NK cells, CD8^+^ cells and type I and II IFN [23]. Notably, synergistic anti-cancer activity was also observed in tumours refractory to NDV oncotoxicity, in keeping with the idea that virus-induced stimulation of the immune system (rather than viral oncolysis) contributes the most to the enhanced therapeutic efficacy [23]. This study provides pre-clinical proof-of-concept that the ability of OVs to induce robust immune responses prepares the stage for CTLA-4 blockade therapy.

The relative timing of OV administration and immune checkpoint blockade is likely to have a great impact on the success of combination therapy. In accordance with the reported peak of CTLA-4 expression around 24–48 h after T cell activation [109], the benefit of CTLA-4 inhibition during VSV therapy decreased when anti-CTLA-4 antibodies were administered 3 days after VSV, and was completely lost after 7 days [108]. The importance of time schedule is also exemplified by a recent study exploring different combinations and regimens of vaccinia virus (VV) and immune checkpoint inhibitors in syngeneic murine models of renal and colon adenocarcinoma [110]. Initiating CTLA-4 blockade concomitantly with systemic VV therapy restricted viral replication to such an extent that combination therapy conferred no therapeutic advantage over VV treatment alone [110]. This effect was associated with an increased number of cytotoxic T lymphocytes recognising viral epitopes in spleens. In contrast, CTLA-4 blockade beginning 4 days after VV injection potentiated VV anti-cancer efficacy, leading to a significant synergistic reduction in tumour growth compared with monotherapy [110]. This synergistic anti-cancer activity correlated with a significant increase in the number of cytotoxic T lymphocytes recognising tumour cell antigens and was mediated by both CD8^+^ and NK cells but not CD4^+^ T cells. The therapeutic advantage of the combination was lost upon IFN-γ depletion, supporting the importance of CD8^+^T cells.

Together, these studies suggest that strong consideration should be given to the treatment schedule when combining OVs with checkpoint blockade. In particular, blockade should be delayed until after the initial phase of virus replication and oncolysis, so that the OV can exert its immune-stimulating activity before the potentiation of this activity by the blockade turns against the virus. The optimal timing for promoting synergistic anti-cancer effects instead of antagonistic anti-viral effects is likely to depend on the OV, immune checkpoint and tumour types involved.

An alternative approach to systemic co-administration of immune checkpoint blockade antibodies and OVs is to directly arm the OV with genes encoding these antibodies. Prime candidates for OV-mediated transduction are anti-CTLA, anti-PD-1 and anti-PD-L1 molecules. This strategy is attractive because it potentially has the advantage of restricting the expression of the antibody within the tumour bed, thus alleviating systemic immune-related adverse events. A proof-of-concept study demonstrated that oncolytic Ad can express fully functional human monoclonal antibodies directed against CTLA-4 while retaining its oncolytic capacity [111]. When the engineered Ad was injected into established human lung carcinoma xenografts, antibodies accumulated at a much higher level at the tumour site compared with plasma [111].

It is possible to reinforce the oncosuppressive activity of MV by inserting transgenes encoding CTLA-4 and PD-L1 antibodies into the viral genome [112]. Expression of the antibodies did not alter the ability of the armed viruses to suppress human tumour xenografts in immune-deficient animals or to replicate in patient-derived human melanoma specimens. In an immunocompetent mouse model of melanoma, which was susceptible to MV infection, significant therapeutic benefits were achieved with both antibody-expressing MVs compared with the unarmed virus. The improved anti-cancer activity correlated with increased levels of activated CD8^+^ T cells and reduced tumour infiltration with regulatory T cells [112]. However, intratumoural injection of an MV vector encoding anti-CTLA-4 antibodies was less effective at prolonging survival than combination therapy with wild-type MV and systemic CTLA-4 blockade [112]. This points towards a possible drawback of using OVs that have been engineered to express immune checkpoint inhibitors, as this approach may lack flexibility with regard to dose regimen and timing. In addition, the tumour-restricted transduction of immune checkpoint inhibitors may be less efficient than their systemic administration in cases where these antibodies target molecules such as CTLA-4, which act primarily in lymphoid organs. Indeed, in contrast to MV expressing anti-CTLA-4, MV expressing anti-PD-L1 antibodies was as efficient as the equivalent combinatorial regimen, possibly reflecting the more peripheral action of the PD-1/PD-L1 checkpoint [112].

### 6.2. Clinical Studies

The clinical translation of combination therapy with OVs and immune checkpoint inhibitors is in progress. Treatments that combine the oncolytic HSV T-VEC with ipilimumab (NCT01740297) or pembrozilumab (NCT02263508) are currently being assessed in early-phase trials for metastatic melanoma. The interim analysis of the phase Ib study of T-VEC plus ipilimumab is encouraging, with objective responses occurring in 41% of patients and a complete response rate of 24% (*n* = 17). Given the reported response rates for T-VEC and ipilimumab as monotherapies (objective response rates of 26% and 10.9%, and complete response rates of 11% and 2%, respectively [5,113]), these data suggest an improvement in efficacy of combination therapy relative to single treatment [114].

## 7. Conclusions

With the recent FDA approval of T-VEC and the progression of other OVs through late-phase clinical trials, this novel class of anti-cancer agents has proven to bring significant benefit to cancer patients. Nevertheless, clinical responses elicited by OVs remain highly heterogeneous, calling for the combination of these viruses with other therapeutics to overcome some of the obstacles that may hamper efficacy. It is becoming increasingly clear that OVs exert their oncosuppressive activity through two major mechanisms: the induction of oncolysis and the stimulation of a robust anti-cancer immune response. An initial period of largely unhindered virus multiplication and tumour cell lysis may be a prerequisite for OVs to act as efficient *in situ* vaccine adjuvants. In particular, we consider that shifting the balance between anti-viral and anti-tumour immune responses in favour of the latter may hold the key to successful oncolytic virotherapy. Pre-clinical studies with many of the OVs presently undergoing clinical evaluation strongly support the use of HDACIs as effective boosters of OV intratumoural multiplication and lytic activity. This class of compounds has been reported to enhance the efficacy of OV infection at multiple levels through the stimulation of viral cell receptor expression, the modulation of viral and cell protein activity and the dampening of innate and adaptive anti-viral responses (Figure 3A). Moreover, some HDACIs have been shown to enhance cancer cell immunogenicity by themselves through the upregulation of MHC and co-stimulatory molecules and by inducing ICD [62].

In contrast, the ability of OVs to induce local inflammation render them well-suited for combination with immune checkpoint blockade. Accumulating evidence shows that stimulating the immune system with immune checkpoint inhibitors can result in a striking, synergistic anti-cancer effect when combined with OVs. “Releasing the brakes’’ on OV-induced anti-tumour immunity through immune checkpoint blockade is under intensive investigation, with promising early results already achieved at both pre-clinical and clinic levels.

As a whole, these data lead us to propose a two-phase approach to oncolytic virotherapy, as depicted in Figure 3. In the first phase, HDACIs are applied transiently, before or concomitantly with the OV, to dampen innate immunity and maximise viral multiplication and spread. Most HDACIs have a half-life of only a few hours and will undergo hepatic metabolisation and subsequent intestinal excretion, thus permitting rapid recovery from immunosuppression. In the second phase, downstream anti-tumour immune responses are enhanced through the addition of immune checkpoint inhibitors. Although this sequential combination strategy is appealing, its clinical translation may require some challenges to be met. In particular, special attention should be paid to the design of dosing regimens that achieve the best compromise between maximising anti-tumour efficacy and mitigating immune-related toxicities. This balance needs to be assessed through extensive pre-clinical testing. The most suitable combinations and optimal dosage regimens will likely depend on the nature of both the OV and the target tumour, necessitating careful optimisation for each individual case.

In conclusion, virotherapy is a very promising anti-cancer strategy. Overcoming barriers to improve the efficacy of OVs can be achieved by combining these viruses with other anti-cancer agents. In particular, combinations of OVs with agents modulating cell permissiveness for virus infection and/or immune responses will undoubtedly come into prominence in the years to come. With FDA approval of T-VEC and the likely licensing of other advanced OVs for use as monotherapies on the horizon, improvement-directed efforts using combinatorial treatments are now expected to come to the forefront of OV clinical translation.
